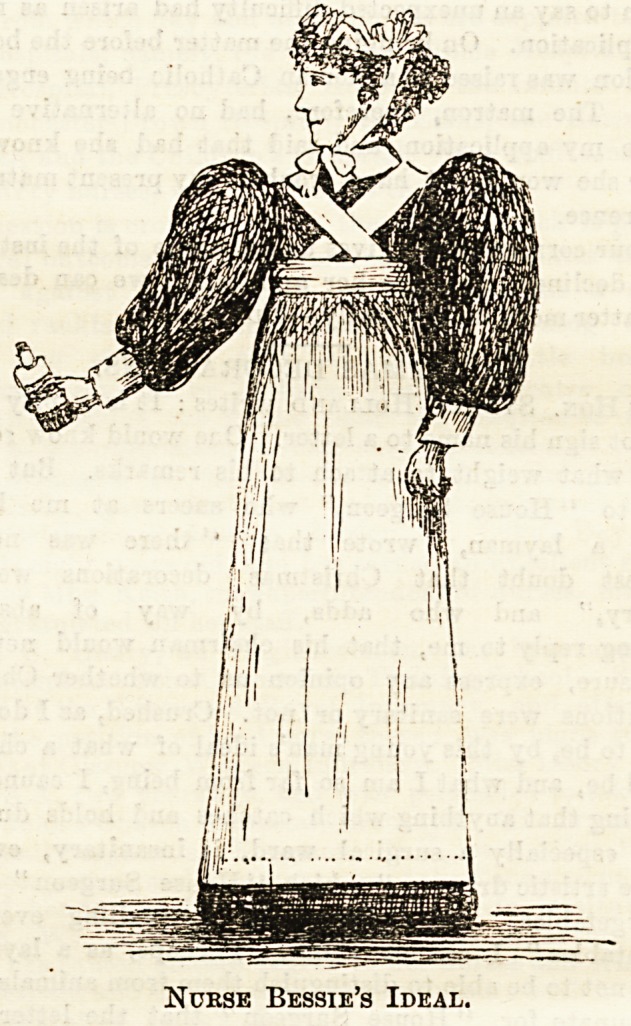# The Hospital Nursing Supplement

**Published:** 1895-12-21

**Authors:** 


"The Hospital" Nursing Mirror.

Being the Extra Nursing Supplement of "The Hospital" Newspaper.

[Contributions for this Supplement should be addressed to the Editor, The Hospital, 428, Strand, London, W.C., and should have the word "Nursing" plainly written in left-hand top corner of the envelope.]

### News from the Nursing World.

### ANOTHER PRINCE.

Our readers are desirous of congratulating their Royal Highnesses the Duke and Duchess of York on the birth of their second son, and rejoice at the good accounts of the progress made by "Our Princess's" daughter-in-law.

### WANTED—BOOTS AND SHIRTS.

The useful gifts which most hospital patients receive on Christmas Day are a cause of much preliminary anxiety to the nursing staff at large institutions, but sometimes capacious bundles arrive opportunely, and help out the matron's store, which dwindles rapidly at this season. But of two special articles there always appears a great dearth, and these are—boots and flannel shirts. It seems as if making a shirt were beyond the power or will of many leisured women, yet these garments are sorely needed by most discharged hospital patients. Boots and shoes are equally necessary and equally scarce, yet there are many useful pairs lying idle elsewhere, which would be thankfully received at a hospital. If our readers would think of this, many a poor man and woman might be for once decently shod. The need for boots and shoes becomes a kind of nightmare to the thrifty poor, whose earnings have been suspended by illness. "I'm going home presently, Sister," said an anxious-looking woman, seated by her own bed; "I hope you'll excuse me bein' a bit late starting." "I don't mind, Mrs. Smith; but I thought your daughter would have fetched you, for you are not very strong." "Oh! I'll get along all right," returned the woman; "it wasn't convenient for my daughter to wait." A sympathetic question from the ward sister elicited the fact that Mrs. Smith's daughter, having brought her some clothes, had now returned home that she might send back by a boy the boots from her own feet to be put on by her mother!

### A CHRISTMAS DINNER.

Last Christmas a handsome present of pheasants from H.R.H. the Prince of Wales furnished an excellent dinner for the patients in a metropolitan general hospital. It was observed that everyone ate with good appetite and left empty plates, and there were none of the jokes which in institutions season a dish of game, and the meal was soon despatched and the plates gathered up. "Well," said an old woman, with a satisfied sigh, "to think as I should live to have such an honour done me!" "What's the matter, Grannie?" asked a nurse. "There ain't nothin' the matter as I knows on. I was only a sayin', just to think of the honour of it! My son will never believe it when I tell 'im as the Prince of Wales went out 'imself to shoot me my Christmas dinner." "But, Grannie," rejoined nurse, mischievously, "I remember your saying that you wouldn't eat pheasant because it was too much like a fowl gone bad!" Grannie shook her head, "True for you, nurse," she replied, "but that didn't refer to birds shot by His Royal Highness for such a poor old body as me."

### TRAINING FOR SOLDIERS' WIVES.

The British Soldiers' and Sailors' Families Association have approached the managers of the Edinburgh Royal Infirmary through the committee with very good result. The latter, it is reported, consented to recommend that a number, not exceeding four, daughters and wives of sailors and soldiers should be received as probationers at the Royal Infirmary, the fee for training being reduced from the customary £25 to £10 in each such case.

### KING'S COLLEGE HOSPITAL.

At King's College Hospital the celebration of Christmas will be restricted to the actual anniversary. Useful gifts for patients, a moderate amount of decoration, a little music, and plenty of suitable refreshments, will all be provided, the interval between dinner and tea being devoted to the reception of the patients' friends, who come in as on ordinary visiting days. The chapel at King's College Hospital is always decorated with great taste and care.

### A WESTMINSTER NURSE.

The inhabitants of Luton are raising a subscription for a memorial to be placed in Westminster Hospital chapel in remembrance of Nurse Florence Allen, who fell a victim to typhoid fever, contracted during the recent epidemic in that town. Not only for her indefatigable personal services is her name revered, but also for the zeal and influence by which she secured the co-operation of many who would not otherwise realised their own ability to assist in an emergency which will long be remembered in Luton. The subscription list is confined to the residents of Luton, which is Nurse Allen's native town, and where, by her own desire, she was laid to her rest.

### HAGGERSTON AND HOXTON NURSES.

A sermon was preached by the Bishop of Stepney at the annual thanksgiving service on behalf of the Haggerston and Hoxton District Nursing Association. Speaking eloquently of the value of district nurses in such a locality, the Bishop drew attention to the urgent need for the services of an additional nurse being secured as soon as funds were forthcoming.

### CHRISTMAS AT CHARING CROSS.

There will be no theatricals in the Board-room at Charing Cross Hospital this year. The patients will have their usual seasonable fare and serviceable gifts on Christmas Day a handsome Christmas Tree, and the students and their friends have a ball at Queen's Hall to anticipate. A supply of tickets for one of the theatres given by a member of the committee has already enabled the nurses to enjoy a little personal pleasure.

### AN ENTERTAINMENT AT WOLVERHAMPTON.

A well-attended "At Home" was recently given by the Ladies' Committee and matron at Wolverhampton Eye Infirmary. An excellent concert took place, and a collection was afterwards made at the doors towards the expense of a disinfecting machine. Last year a similar entertainment resulted in £50 being raised for the extension of the heating apparatus.

### QUEEN'S NURSES IN LINCOLN.

The Lincolnshire branch of the Queen's Jubilee Institute held its annual meeting at the Town Hall, under the presidency of the Mayor. Several additional members were placed on the committee. Mr. Younger, M.P., was re-elected president, and Lady Winchilsea, the Hon. Blanche Dundas, Miss Sewell, Mr. Burdett, and the Mayor were appointed vice-presidents.

### TEACHERS' HOME OF REST.

The Teachers' Home of Rest at Hastings was established by the Sunday School Union, whilst Clacton and Bournemouth were chosen for the Children's Convalescent and Holiday Homes. These establishments have been heartily appreciated by a large number of guests who have visited them during the past year. Through the winter also every facility is offered by the Union to delicate teachers and children to gain renewed health. In summer the houses are constantly full, but there are a few vacancies just now of which particulars can be learnt from Mr. Clement, hon. secretary, 56, Old Bailey, London.

### THE PETER BROUGH NURSES.

The third annual report issued by the Peter Brough District Nursing Association, Paisley, shows that excellent progress has been made in the last year. The staff consists of a superintendent and five nurses affiliated with the Queen's Jubilee Institute, and another nurse will be engaged at an early date. The trustees of the Peter Brough Charity have purchased a site for a permanent nurses' home, which they hope will be satisfactory in every way. The number of patients sent to the Convalescent Home at West Kilbride during the past year is reported as twenty-one. The class of cases which have been under the care of the Peter Brough Association show the necessity of thoroughly efficient nurses being employed for the sick poor in their homes.

### HAVERFORDWEST INFIRMARY.

A children's ward has been established at the Pembridge and Haverfordwest Infirmary by the policemen of the county. It contains eight beds, thus bringing the total number of becs in the infirmary up to forty. A further extension is being carried out by Sir Olver Scourfield, Bart., who is erecting a new wing in memory of the late Lady Scourfield, who was a very good friend to the institution, in which she took much interest.

### LOST PROPERTY.

When the inventory is taken at Bristol Lunatic Asylum it must expose remarkable deficiencies in the stock according to the recent disclosures of the Stapleton Urban District Council. The clerk to this body stated that a sewer became blocked, and the cause of the obstruction was shown to be scrubbing brushes, wearing apparel, felt hats, stockings, and a large quantity of dusters and rags, some of which bore the stamp of the asylum. Surprise having been expressed by the urban authorities at it being possible for such things to gain access to drains, it has been decided to take steps to prevent a recurrence of the nuisance.

### OUR AMERICAN SISTERS.

The nurses' home at the New York Hospital is one of the most complete in the United States. All the nurses have separate rooms, and to every two is allotted a sitting-room situated between their sleeping apartments. A specially good tone exists in this training school, and the nurses are notably nurse-like in appearance and manner, the course of instruction for the probationers being a most thorough one. It is with particular regret that we record that serious illness has overtaken the admirable lady superintendent, Miss Irene Sutliffe, to whom the training-school owes so much of its success. When Miss Sutliffe and her sister, the superintendent of Long Island Hospital, visited England in 1892, they inspected several of our hospitals and made many friends, to whom the tidings of our American Sisters' present trouble causes infinite regret.

### NEW NURSING STAFF FOR JOHANNESBURG.

The nursing at Johannesburg Hospital has been recently subjected to somewhat severe criticism, the committee contending that evils could not be existent unless attended by formal complaints. It appears, however, that many of the doctors have long been dissatisfied with the insufficient skill and inadequate numbers of the sisterhood constituting the nursing staff, but the older Johannesburg residents have naturally been unwilling to call attention to the deficiencies of those who worked for them before either hospital or town attained to their present size and importance. The committee, after much discussion, were prevailed on to consent to the introduction of a proper system of trained nursing in the hospital, and sent over their resident medical officer to select some thirty nurses in England. These have now been chosen by the kind help of an experienced London matron, and not one of those selected has had less than three years' thorough training, and most of them considerably longer hospital experience. If the superintendent who will be placed at the head of such an excellent staff proves herself competent to manage and organise a first-class training school at Johannesburg, that hospital need not in future fear comparison with any in South Africa. The proposal of the committee to retain a proportion of the old nurses must surely be rescinded when they realise the impossibility of partially-trained religious sisters working harmoniously under the new regulations. Such an attempt would be not only unfair to the new superintendent, but also to the sisters whose goodness and gentleness have won for them regard and esteem.

### EMPLOYMENT FOR IDLE FINGERS.

Basket work as an occupation for chronic invalids is suggested by one of our correspondents. It might equally well be recommended as an interesting employment for convalescents, especially for boys and girls, whose restless fingers would find its novelty a diversion.

### SHORT ITEMS.

The Cardiff Guardians voted an annual subscription of £10 to the Victoria Nurses' Institute at Penarth, which had nursed many outdoor paupers during the course of the year.—The first annual report of the Penryn and St. Gluvias District Nurse has been issued, and shows that the services of Nurse Constantine have been greatly appreciated.—The successful bazaar in aid of the Barry Nursing Association and Accident Hospital extended over three days.—The second annual report of the Bath Ladies' Royal United Hospital Working Association shows a satisfactory financial position, and a total of 808 gifts to the hospital. The association has 335 members.—A successful bazaar has been held in aid of the Batley and District Cottage Hospital.

### Lectures on Nursing.

By a Superintendent of Nurses.

### I.—INTRODUCTORY.

People talk about the noble work of nursing, and some of the worst nurses I ever met used this phraseology most glibly. Now let me beg of you to put away all cant in speech and thought as well as all romance; do not picture yourselves as angels of mercy giving cups of cooling drink and surrounding yourselves with halos of glory.

Hospital work is a stern reality, not a game to be played at; it needs strong, brave women for it, and should call into activity all that is best in woman's nature—patience, unfailing kindliness, thoughtfulness, self-reliance, obedience, pity, sympathy (the putting of one's self in the place of the sufferer), in short, self-sacrifice.

All this means a great strain upon a woman, and unless her health is good it is unwise for her to undertake duties for which she is clearly unfitted, even though she may gain admittance into a hospital.

Everyone taking up nursing should do so in an earnest spirit, desiring to "do noble deeds, not dream them all day long," praying to be kept pure and gentle in thought and true in word and deed. Those of you who have entered hospital work in the right spirit will find an immense amount of happiness in store for you; for whatever drawbacks this life has, and it has many, it has pleasures which no other life can yield. At first you will feel inclined to think this cannot be the case. Tired and footsore, home sick and lonely, you will frequently be tempted to throw up the whole thing, and fancy you have made a great mistake in attempting to nurse the sick.

Not only in the wards will you find your need of patience, self-control, and unselfishness, but also in the family life of the hospital nursing staff. You may meet with those who differ from you on matters about which you have thought there could be no two opinions. Faults may be found in you that were quite unsuspected by you before. You may feel an inclination to say sharp and bitter things. You may meet with those whose religious opinions are not at all like your own, and who will be very ready to sneer at any inconsistency on your part. You will mix with some who grumble at everything and do their utmost to spread abroad a spirit of discontent; with others who love to gossip and make much of petty hospital scandal. All this is very trying when met with. You are made to feel that you are indeed no longer at home. Let your life speak for you. "If ye serve the Lord Christ" try to think and do what would please Him in even the smallest thing. "Whatsoever things are true, whatsoever things are honest, whatsoever things are just, whatsoever things are pure, whatsoever things are lovely . . . think on these things." Erskine says, "Life is not divided into religious and secular parts; all should be religious . . . the world is a temple, and the business ought to be the services of the temple."

Set the right always before you, and never sanction wrong in any shape or form in yourself or others. If you ever hear a fellow nurse saying anything which may injure or grieve you, go to her at once and try to set matters straight. Put as kindly a construction on everything as you possibly can, and try to help your fellow nurses. Be kind to any new probationers, who may be feeling lonely and unhappy. It will no doubt seem strange to you to have to do many things which, as nurses, it is desirable you should do. You meet people you would usually associate with as equals on a different platform altogether, and your connection with them is purely official. You must be prepared to treat with deference those who are officially your superiors, and to give those in authority implicit obedience. "Who rules o'er freeman must himself be free," and certain it is that those who have never learned to give obedience will never command it themselves.

Your connection with the house surgeons and dressers is purely official, and you should never forget this. Any thoughtlessness on your part or indiscreet action may result in your becoming the talk of the hospital. You are not in a position to know the men you meet in your work, so you have need to keep up rigidly your self-respect. Your intercourse with them should be confined to matters connected with your duties, for you are working in an institution where all you say and do, and all that you yourself are, have a wide influence for good or evil. Think, then, of your individual responsibility.

Now, as to dress, I cannot but think outdoor uniform is a mistake under ordinary circumstances. It seems to me it is better for anyone living in a hospital, when off duty, to lay aside everything suggestive of sickness; and the mere changing of a dress is a relief, quite worth the very short time spent in doing so. As to your indoor dress, you are expected to be always in uniform. Your dress should be short enough to clear the ground. Your caps, collars, and cuffs should be as clean as possible. You should be very careful to avoid getting into a slovenly way of putting your things on, pinning apron or dress if a button has come off; putting your cap on so that it looks as if it did not belong to your head, for untidy hair always gives a nurse an untidy look. Wear sensible shoes, with low heels. You can hardly imagine how annoying the tap-tap-tap of high heels, or the squeaking of boots, can be to sick people. If you have never been ill yourself or accustomed to illness you can but faintly understand the irritability of an invalid whose nerves are so disordered that the slightest annoyance of this kind may give untold pain. Therefore learn to feel with your patients.

You want to secure your patient's restfulness. Now an untidy and thoughtless nurse will never conduce to restfulness. The patient will be conscious of disorderly surroundings if the nurse has untidy hair, her cap falling off her head, a long dirty tail to her dress, or an apron soiled or torn. Try to keep yourselves as clean as possible whilst about your work.

In sickness some of the senses are greatly sharpened. The least smell of cooking produces nausea, and the sense of hearing is very keen. I often think the chatelaine abomination must be most exasperating to patients. Avoid leaving a door unlatched so that it keeps up a perpetual nagging sound. Try to avoid everything that is likely to produce uneasiness or annoyance to those under your care. This you can accomplish only by habitual thoughtfulness, and it rests with you whether you refresh and gladden those about you or cause discomfort and depression.

### Appointments.

Ormskirk Cottage Hospital.—Miss Isabel S. Matheson has been made Matron of this hospital. She was trained at the Northern Infirmary, Inverness, and afterwards worked at Blackburn Infirmary. We congratulate Miss Matheson on her appointment, and wish her every success in the post for which she has been selected from a large number of candidates.

Wallsend and Willington Quay and Howdon Joint Hospital Board.—Miss M. Wooll, who has been appointed Matron of this hospital, was trained at the Cheltenham General Hospital. She worked at the Isolation Hospital, Stapleford, and was subsequently made matron and superintendent of the Hertford and Ware Joint Hospital. We congratulate Miss Wooll on her appointment, and wish her all success in her new post.

Dreadnought Seamen's Hospital, Greenwich.—Miss Hall, who for nearly two years has been matron of the Branch Seamen's Hospital in the Royal Victoria and Albert Docks, has been promoted to be Matron of the Dreadnought Seamen's Hospital. Miss Hall was trained at Guy's Hospital, and was subsequently assistant matron at Dulwich Infirmary. We congratulate her on her appointment, and wish her continued success.

### "A National System of Notification and Registration of Sickness."

(Communicated.)

The above is the title of an interesting paper read before the Royal Statistical Society on the 17th inst. by Dr. Arthur Newsholme, Medical Officer of Health of Brighton. The lecturer first gave an account of the various attempts at sickness registration. This originated in a system of death certification and registration. The office of Registrar-General of Births, Deaths, and Marriages in England was first established in England in 1836, and the first report appeared three years later, and on this have been built up the great sanitary improvements which have already been made by arousing attention to and interest in the subject.

The next step in advance was the discovery that a mean registration of deaths gave a very imperfect idea of the prevalence of disease, because some are never fatal, but cause incapacity and great suffering. It was found that no ratio can be fixed between sickness and mortality, because mortality is not necessarily a correct index of the morbidity of a community, and occasionally the highest ratio of sickness is associated with a favourable death-rate. From the standpoint of the common health, sickness is more important than death, or, as Charles Dickens says, "It concerns a man more to know the risk of the fifty illnesses that may throw him on his back, than the possible date of the one death that must come. We must have a list of killed, and of the wounded too."

The first local Act for enforcing compulsory notification of the chief infectious diseases came into opperation in September, 1877, at Bolton, and other towns soon followed the example, though it was not till 1889 that the Infectious Diseases (Notification) Act was passed, and was quickly adopted by both urban and rural sanitary authorities throughout the land. Dr. Newsholme says, however, that it "has been proved that compulsory notification of infectious diseases has in special instances increased the prevalence of those diseases." Notification, however, has been of undoubted value in leading to immediate preventive measures of a most successful character, and in other instances to force the most radical reforms upon even unwilling sanitary authorities. Since 1889 there has been great activity in providing isolation hospitals, which, if they have uot always diminished the prevalence of infectious diseases by removal of those who were first affected, have provided better and more successful treatment for those patients who have been removed, and have lessened mortality, and have greatly reduced the malignancy of such diseases as scarlet fever. This is due to the fact that infectious diseases are now manly treated in large airy wards, where the virus of the disease is not concentrated by unfavourable surroundings, and where the patients are skilfully nursed. Other benefits that have followed compulsory notification are more efficient means of disinfection, the removal of those insanitary conditions to which in some instances maladies owe their existence, and the steady education of the public in hygienic matters. Moreover, a mass of information is being accumulated regarding the causes on which depend the prevalence of various infectious diseases, which knowledge must ultimately increase the success with which epidemics are treated.

Unfortunately, there is still much difference of opinion as to which infectious diseases should be notifiable. Dr. Newsholme would make all such notifiable, though he recognises that the expense involved would be a serious consideration, as also the question of the payment of the medical officer.

The lecturer next gave a short account of the system existing in various foreign countries respecting notification, and pointed out how much more complete their methods were than ours, and that all this work was done gratuitously.

Since accurate knowledge of sickness, and of its degree of incidence in relation to sex, age, occupation, housing, and locality, &c., must precede all national preventive measures, Dr. Newsholme would like to see the following measures adopted:—

First, all cases of sickness occurring amongst the parochial poor in each district should be periodically reported to the medical officer of health, and tabulated statements forwarded to a central office, where such statistics should be analysed, summarised, and published.

Secondly, all cases of sickness, whether out-patients or in-patients, at hospitals (general and special) and at public dispensaries should be reported weekly or monthly to the medical officer of health, and by him forwarded to the central office, to be there treated like pauper statistics.

Thirdly, all friendly and sick insurance societies of every description should be required to furnish weekly or monthly returns of the number of new cases of sickness in their experience, classified according to a specified schedule, and a yearly statement of the total subscribing number of members, classified according to age.

Fourthly, an attempt should be made to obtain accurate returns of sickness in the great industries.

This will be greatly facilitated when the new Factory Act comes into force on January 1st, 1896, and no doubt it will then be possible to secure both an accurate return of the number of men employed in each industry and also of the cases and causes of sickness, each classified according to age.

Fifthly, the schedule of notifiable diseases should be widely extended, and classified as those compulsorily notifiable (*a*) within twenty-four hours and (*b*) weekly. The list of the latter should be greatly increased, and include pneumonia, rheumatic fever, phthisis, and other diseases, such as lead-poisoning, &c.

### St. George's Hospital.

### REOPENING OF THE CHAPEL.

An interesting ceremony took place at St. George's Hospital last Tuesday night, on the occasion of the reopening of the chapel by the Bishop of Marlborough. A chancel has recently been added, and other alterations and improvements made, not the least among them being the handsome stained glass windows given by the nurses and their friends. The Bishop, preceded by the long line of clergy and choir in their white robes, passed up the aisle between the neat-looking rows of nurses in their spotless caps and aprons. A short special service with appropriate Psalms and hymns was heartily rendered, on conclusion of which the Bishop gave an address. Taking for his text (St. John v. 6), "Wilt thou be made whole?" he dwelt in eloquent terms on the spiritual ministrations that go hand in hand with medical and nursing skill, and the mystical meaning of the Bethesda in the parable, and the opportunities for good which a hospital affords. The hymn, "Light's Abode, Celestial Salem," brought the bright little service to a close. The Bishop was afterwards entertained at supper in the board-room of the hospital, Mr. Timothy Holmes, treasurer, presiding. Several of the governors and friends of the hospital were present, and a very pleasant evening was spent. In a few well-chosen words Mr. Timothy Holmes, on conclusion of the repast, thanked the Bishop for his presence among them on the occasion, to which the Bishop responded in an admirable speech, enlivened with witty reminiscences and enriched by classical quotations. Thanks to the untiring energy of Mrs. Coster and Mr. Todd, no efforts had been spared to make the arrangements as perfect as possible, and it was with real regret that the inevitable adieux were ultimately said, and a most interesting evening brought to a close.

### Christmas in Hospital.

### INTRODUCTORY.

The prospect of the first Christmas Day in hospital is viewed with mixed feelings by most people, and new patients are often heard to remark in very despondent tones, "I suppose I'll have to stop in over Christmas." To everyone possessed of a home, that home is the spot where Christmas should naturally be spent, and it is, therefore, with some feeling of personal injury that the sick man (or woman) finds himself detained in a ward towards the end of December. But seldom indeed does this feeling predominate when Christmas really comes. The pleasant preliminaries show that something may be anticipated, the whole atmosphere of the hospital being pervaded with a promise of good things to come. Nurses comment on "What we did last year," and in the intervals of work they relate anecdotes of past Christmases, to which the new patients listen with keen but silent interest.

The realities of Christmas Day are always better than the anticipations, and few patients have cause to regret their detention in hospital. There is an increasing tendency to condense Christmas doings into the one day instead of allowing the entertainments to extend over Boxing Day, and this plan has advantages and drawbacks. Amongst the former may be reckoned the shortened period in which the ordinary routine of work is interfered with, and in the latter the fact—distasteful to some—that the religious aspect of the festival is crowded out by the many things which are pressed into one short day.

The large ward entertainments so widely favoured some years back have been greatly modified of late, committees and hospital staffs having become fully alive to the danger of the entertainers losing sight of the primary object of their presence, *i.e.*, the pleasure of the patients. When the provision of any kind of amusement necessitates the presence of too many performers and their admiring friends the wards may become inconveniently crowded, and the head nurse be distracted by the task of looking after strangers as well as patients. Should the latter include serious cases there are other obvious disadvantages to fear. So long as the well-being of the patients is the first consideration entertainments cannot fail to give unalloyed pleasure in the wards.

Perhaps the most practical point about Christmas Day is the present provided for each patient. Cards, cake, and crackers sink into insignificance before the substantial benefit of a flannel shirt, a warm shawl, or a couple of pairs of good socks or stockings. Only those who have witnessed the pleasant sight can realise the extraordinary enjoyment a present gives to those unaccustomed to gifts. The acceptable offering is looked at frequently during the day and carefully packed up again after each inspection.

In hospitals possessed of a suitable room entertainments are often provided expressly for the nurses at some date nearly connected with Christmas Day, which is itself rightly devoted to the service of the patients. In other hospitals the treat to the nurses takes the form of a supply of theatre tickets provided by members of the committee to be used by small parties of nurses on successive evenings, a good concert or play being usually found more popular with nurses than a general hospital gathering of those who work daily side by side.

### Within the Hospitals.

Christmas at St. Thomas's Hospital is made a very pleasant time for the patients, who all take great pride in the tasteful decorations which are to be seen in most of the wards. Everything is done to make the day a happy one, and the entertainment of the inmates does not end with it, for on one of the following days the Nightingale probationers go round with the home sister and sing carols in each ward. Many old workers come back for the occasion, and it is difficult to imagine a prettier scene than that presented by the orderly wards in the soft light of the coloured lanterns and shaded lamps, and the group of singers standing in the centre. Another enjoyable gathering takes place annually at St. Thomas's when the students arrange a capital concert, to which they invite the nursing staff and their friends.

Every patient has a useful gift at Westminster Hospital, and to the preparation of these much attention is given, for the suggestion that "anything" will do for the poor finds no favour there. The shirts for the men are strong, warm, hand-made garments, just what convalescents need when the day of departure draws near; and gifts for the women are of an equally practical description. The dolls dressed for the sick children by two little "chronics" in the incurable ward are so pretty and dainty that the visitor cannot wonder at the pride with which the two small workers bring them forward. They are deftly wrapped up to keep them fresh till the last moment, and there is something very touching about the enthusiasm with which the permanent invalids provide pleasure for those whose illness is often of a temporary though acute character.

The word chronic seems to take us on to the Hospital for Incurable Children, 2, Maida Vale, where for weeks past the festival has found its way daily into the conversation of the little ones. They never tire of the subject, and go to bed on Christmas Eve full of anticipations. Every leaf in the decorations which friends come and put up for them is a joy to these helpless children. "Christmas Day's like Sunday," one of them sums up shrewdly, "only its better 'cos of the presents." As for cards, why, they have brought a foretaste of Christmas for days past! Then there's a real Christmas service, the children tell you, and plenty of hymns are sung by those sweet, well-taught little voices. Good friends provide more than one Christmas tree, and the seasonable treats for the children are prolonged as much as possible.

At the Temperance Hospital Christmas Day is devoted to the patients, the nurses' entertainment taking place at the beginning of the New Year, and the Christmas tree at the end of the old one.

At Guy's Hospital the entertainments in the various wards are permitted during the whole of Christmas week, and the wards are gaily decorated, Useful presents for the patients form here, as elsewhere, one of the best features of the festival to inmates whose experience of gifts is generally pathetically small. The mothers nursed in their own homes under the maternity charity of Guy's Hospital have also Christmas boxes of much-needed comforts, these including some tea and sugar.

The women students at the Royal Free Hospital provide two pleasant evenings for patients and nursing staff in the current week. They have theatricals and music in the board-room for those who are able to be present, whilst for the patients who cannot leave their beds ward entertainments are provided by the students. Christmas Day is made pleasant by the nursing staff, and the festive ward teas they provide are a pretty feature of the occasion and popular alike with male and female patients.

St. Bartholomew's Hospital is fortunate in the possession of the fine hall which facilitates the arrangement of entertainments and receptions, in addition to the usual Christmas Day doings in the wards.

At the London Hospital the whole of the ward entertainments take place on Christmas Day, and they are varied and excellent, many outside friends devoting the evening to giving pleasure to those who spend Christmas Day in hospital. The children's Christmas tree entertainment is to take place on the 30th inst.

A magic lantern entertainment has already been enjoyed by the patients at the Children's and Women's Hospital, Waterloo Bridge Road, and King's College Musical Society provides a good concert there. The Christmas tree will be held on December 30th.

At the Great Northern Central Hospital, in Holloway Road, the patients are regaled on Christmas Day with an excellent dinner, and have presents, carol singing, &c. Later on an entertainment is arranged for them, and the Ladies' Association kindly co-operate with the staff in providing a seasonable tea.

The patients at the Brompton Hospital for Consumption have turkey and plum pudding served to them, the turkeys being presented by three or four friends. The sisters and nurses have their respective Christmas dinners on three days, a third of the staff being present on each occasion. Theatre tickets are also taken for their benefit. A grand entertainment is also provided for the patients, nurses, and servants in the beautiful concert-room on December 31st, and each person receives presents from the great tree. After this has been stripped a concert takes place, arranged by the resident medical officers and their friends.

Preparations for Christmas are going on at the Middlesex Hospital, where piles of neat parcels already contain the patients' presents duly labelled. The chief entertainments take place in the board-room, where a magnificent Christmas tree is laden with useful presents for those well enough to go there. For patients confined to bed a portable "bran pie" makes its appearance in each ward, and is carried from bed to bed.

Each patient at the Chelsea Hospital for Women is presented by the committee with some useful article of clothing and a Christmas card, and is allowed to invite a friend to tea. The Ladies' Committee provide Christmas dinner, consisting of turkey, plum pudding, and dessert; and in the evening the nursing staff sing carols in the wards. The Ladies' Committee supplie presents for the nurses and household staff, and also their Christmas dinner. On January 11th a grand entertainment will be held for the patients, the tea being followed by the performance of a ventriloquist. A handsome Christmas tree is to be laden with gifts for the patients, nurses, and household staff.

The Christmas entertainment at University College Hospital will take place on Monday, December 30th, from half-past five to eight p.m.

"The Follies" paid a recent visit to the Victoria Hospital and entertained the children and their friends with a pretty and varied selection of songs, dances, and instrumental music. "The Follies" were effectively dressed in the Pierrot costume, carried out as regards half the ladies in scarlet and white, the other half and the gentlemen being in white and black; and it would be difficult to imagine a more attractive entertainment than this little company provided.

### District Nurse at Cockermouth.

The *West Cumberland Times* contains a report of the case of Nurse Victoria Murray (50), who was fined 15s., including costs, on the 9th inst., by the magistrates, for being drunk and disorderly at Cockermouth. She had obtained the post of district nurse before the committee had authenticated her testimonials. A summons on a charge of fraud in obtaining a situation as nurse with the Cockermouth Nurse Committee was at the same time withdrawn on Mrs. Victoria Murray's conviction for drunkenness. Mrs. Murray did not appear.

### Our American Letter.

(Contributed.)

A luncheon, to which over a thousand guests sat down, followed the opening of the Chicago Hospital in the Forty-ninth and Drexel Boulevard, and a large reception was held there in the evening. The hospital is well equipped, and contains sixty private rooms for paying patients, a large ward for those who are only able to contribute small sums, three operating rooms, and the usual administrative accommodation.

The Homœopathic Hospital at Baltimore is to receive considerable structural additions next year, and the last annual financial report of the institution is a satisfactory one.

The projected farm colony for epileptics is likely to become a practical reality at an early date, as a charter has been secured for it under the title of the Pennsylvania Colony Farm for Epileptics. An offer to erect suitable cottages has been made by a liberal citizen, conditional on a suitable site being secured by New Year's Day. It is proposed that a hospital shall form one feature of the new settlement.

The Wisconsin Training School is really a kind of central home, from which day and night nurses are supplied to eight small hospitals, namely, the National Home Hospital, Wisconsin Hospital, Presbyterian Hospital, the Infants' Home, the Manchester-Brown Hospital, the Elans, the Emergency Hospital, and the Children's Hospital. The staff consists of about sixty nurses, who all sleep at the home.

A question is cropping up in the States which is perhaps a natural outcome of nurse training, for graduates are protesting against a certificate gained only in a special hospital ranking with that issued by a training school. It is also objected that at present little hospitals with only, say, 12 or 14 beds, grant certificates, on the strength of which the pupils claim the position of trained nurses at the completion of their hospital course.

The managers of St. Luke's Hospital Training School, Utica, N.Y., gave their first public entertainment in connection with the graduation class on St. Luke's Day. The school is eight years old, and has earned an excellent reputation, and Miss Keith, the superintendent of nurses, may well be congratulated on her staff.

Miss S. Henry, an Englishwoman, has been appointed superintendent of the Connecticut Training School. She was trained at the Liverpool Royal Infirmary, and afterwards filled the position of head nurse there; was night superintendent at Glasgow Western Infirmary. Miss Henry visited the U.S.A. two years ago, and was persuaded to take work in St. John's Church Hospital, Brooklyn. The organisation of a small hospital at Kingston, N.Y., was Miss Henry's next occupation, and the managers of the Connecticut School invited her to transfer her services to their institution on Mrs. S. W. Quintard's retirement. The departure of the latter has caused sincere regret to her nurses and friends in Connecticut, and she takes many good wishes with her to her new work at St. Luke's, N.Y.C.

The School Registry for Nurses, a co-operative scheme formulated by the New York City Training School some five years ago, has met with steadily increasing success. It is exclusively for nurses trained at this school. By this scheme they are enabled to take up private nursing and live at a central home, and receive their own fees. The rules are simple, and Miss Louise Darche is to be congratulated on the success of this as of many other well-considered plans originated by her.

The third annual convention of the Society of Superintendents of Training Schools will take place at Philadelphia on Feburary 12th and 13th next year. The subjects to be discussed are as follows: (1) A National Association for Nurses and its Legal Organisation. (2) A Statistical Report of Working Hours in Training Schools. (3) Training School Registers. (4) Limitation of Pupil Nurses' Duties in Caring for Male Patients. (5) Should Undergraduates be Sent Out to Private Duty? (6) Uniforms. The president of the Society of Superintendents is Miss M. E. P. Davis, University Hospital, Philadelphia, to whom inquiries respecting it should be addressed.

### Everybody's Opinion.

[Correspondence on all subjects is invited, but we cannot in any way be responsible for the opinions expressed by our correspondents. No communications can be entertained if the name and address of the correspondent is not given, or unless one side of the paper only be written on.]

### DISQUALIFICATIONS.

"A Trained Roman Catholic Nurse" writes: I would like to know why Roman Catholics are not admitted into certain London and provincial nursing institutions? What is the object in disqualifying a Roman Catholic? Seeing an advertisement in The Hospital of a vacancy likely to suit me, I asked for a form of application and rules. They were filled up by me and duly posted. Two or three days elapsed before I received a letter from the matron of the institution saying that she had written to my present matron for my testimonials, and had received a favourable reply, and as she thought a personal interview desirable, she would come to London and make final arrangements. I considered myself as good as engaged, but received another letter from the matron to say an unexpected difficulty had arisen as regards my application. On bringing the matter before the board an objection was raised to a Roman Catholic being engaged as nurse. The matron, therefore, had no alternative but to decline my application, and said that had she known this earlier she would not have troubled my present matron for a reference.

[If our correspondent gives us the name of the institution which declined to receive her application we can deal with the matter more satisfactorily.—Ed. *T. H.*]

### CHRISTMAS DECORATIONS.

The Hon. Sydney Holland writes: It is a pity a man will not sign his name to a letter. One would know so much better what weight to attach to his remarks. But let me reply to "House Surgeon," who sneers at me because I, as a layman, wrote that "there was not the slightest doubt that Christmas decorations were insanitary," and who adds, by way of absolutely crushing reply to me, that his chairman would never, he feels sure, express any opinion as to whether Christmas decorations were sanitary or not. Crushed, as I doubtless ought to be, by this young man's ideal of what a chairman should be, and what I am so far from being, I cannot help repeating that anything which catches and holds dust in a ward, especially a surgical ward, is insanitary, even the "more artistic drapery" which "House Surgeon" prefers to vegetables. Why he minds my calling evergreens "vegetables" I cannot think. Perhaps, as a layman, I ought not to be able to distinguish them from animals. It is unfortunate for "House Surgeon" that the letter which precedes his from an "Old Nurse" speaks of the "dusty job" of taking down the decorations. I entirely agree with an editorial remark of your own that the same objection may be made to pictures. I was very properly reminded of this by an American specialist who came round the Poplar Hospital wards not long since, and strongly objected to the pictures as being dust collectors. But with pictures the question arises, as with these decorations, whether their moral effect in making the inmates happier is worth the undoubted risk of having them there. I do not feel quite sure that it is, if I am to believe all I hear from many members of the medical and surgical profession. That is for every committee to decide for their own hospital. But I did not in my letter attach very much importance to the sanitary argument. I think decorations chiefly objectionable because of the labour and trouble connected with them, which does not fall on me or on the committee, but on women who are working quite hard enough as it is. Of course I know that no nurse is forced to help to decorate, but where decorating is done at all everyone does help because no one likes to be churlish. It is easy to work up an hysterical cry about the "joys of Christmas," "patients' thanks with tear-dimmed eyes," "beauty into unlovely lives," "nothing to you who have luxurious homes," and so on. In fact, it would be easier to write on this side because sentiment is with one. Sentiment apart, many a sister and nurse would be grateful if committees would say "No decorations." The Hospital put the case admirably last week under the heading, "What Christmas Means to Hospital Officials." But this need not mean an entire exclusion of every sprig of holly or mistletoe, if that dangerous vegetable is admissible into a hospital at all. A good Christmas dinner, a conjurer or concert, presents to all the patients, a Christmas tree for the children, will be our Christmas at Poplar, and I am sure we shall be able to make our patients happy, even if we cannot reduce them to tears. The house surgeon writes that the remark as to expense comes "artlessly from one who is appealing for £3,000 for isolation accommodation for a service of 60 to 80 beds." There he shows inaccuracy and ignorance. I made no complaint of the expense as falling on the hospital funds, but only agreed with a passage in The Hospital that the expense often fell on the nursing and medical staff, which was most objectionable. My committee will spend as much on Christmas treats as heretofore, but the money will be better spent. How can we in London build and furnish an isolation block to accommodate men, women, and children, of at least six beds, with rooms for four nurses, one servant, small kitchen, bathroom, &c., for a sum much under £3,000? We have 65 surgical accident beds to provide for, of which 20 are occupied by women and children.

[No one has questioned our correspondent's will and power to give a happy Christmas to the inmates of the little accident hospital for which he has already done so much. Our other correspondents, who have also claimed a right to be happy and to give happiness in their own way, may surely be allowed it? When experienced working nurses protest against a sweeping condemnation of their cherished holly wreaths, it seems to us there is no more evidence of hysteria in their kindly words than in those remarks which provoked this defence of old customs.—Ed. *T. H.*]

### NIGHT AND DAY DUTY.

"An Old Time Nurse" writes: I cannot let the question of ovariotomy nursing pass without saying a word about it, for during sixteen years I nursed under the greatest surgeon of the day. Half of this period I spent in a charming little hospital, where I took case after case, often as many as three and four in the month. For eight years after I was doing private nursing, and had some very bad cases, and never once had the help of another nurse. I took my meals and slept in the same room with the patient, unless I had one that communicated with the patient's. In fact, I never left my case for the first week. The work was very hard at first, but I was always in good families, so had a good time on the patient's recovery, often getting trips to the seaside and other interesting places. I had many handsome presents, and, best of all, the gratitude and thanks of the family. I never like help, always preferring to take entire charge of the case. Having given up nursing for a few years, I find it very hard to get any now; and should some of my much-loved work fall again to my lot I should gladly take it *without help.* I must say that self was never thought of, the patient being first in everything in my day.

### COMFORTS OF NURSES.

"Another Working Woman" writes: The article on the "Comforts of Nurses" in your issue of December 7th has interested me very much. I quite agree with the writer that nurses are too much talked about, and I hope that it will ultimately prove to have benefited them, but I think it is doubtful. Primarily the hospital nurse exists to help her fellow-creatures in time of sickness; but help should be mutual, therefore her fellow-creatures should help her. Granting that she enjoys many comforts and blessings, is it not right that it should be so? She does work that the majority of art students, typewriters, and governesses shrink from. Is there any work which demands so much patience and unselfishness from workers as that of nursing? Medical and nursing care, I think, may be discounted, for should a nurse require this often, she will certainly be told she is not strong enough for the work. The sick nurse only receives the same care and attention that is given to any patient admitted to the wards of a good hospital. If after several years' work she falls ill, it would be very hard if she could not claim the attention the nurse had helped to give to so many. Unquestionably the nurse has to practice all the virtues that, as a rule, her patients cannot be expected to possess. She has special work to do and must be helped to do it; the only practical way to help her is to shield her from some of the worries of everyday life.

[Our correspondent fails to see that it is just because she receives the nursing and care of which she appears to think so lightly, that her lot is better than that of the governess, typist, or clerk. They, too, fall ill; they, too, break down, and have neither "sick pay" nor free nursing. Nor is their calling surrounded by the halo which somehow still attaches to the professional nurse.—Ed. *T. H.*]

### Minor Appointments.

Rainhill Asylum.—Miss Arrowsmith, who was trained at the Royal Infirmary, Liverpool, has been appointed Head Nurse (assistant matron) at this asylum. We wish her success in her new work.

Chelsea Infirmary.—Miss Elizabeth Davies has been appointed Assistant Matron at the Chelsea Infirmary. Miss Davies was trained at Chelsea, and subsequently rose to be charge nurse and night superintendent, which post she has held up to the present time. Her experience and qualifications are of a wide and valuable nature.—Miss Ellen Norton Colman has been appointed Night Superintendent at the Chelsea Infirmary. Miss Colman was trained at the London Hospital, and worked subsequently for two and a half years on the staff. Her testimonials are excellent.

### Notes and Queries.

### Queries.

(57) *Training.*—Can you give me address of a small maternity hospital which, I believe, is somewhere near Commercial Road East? Do they take pupils there?—*Monthly Nurse.*

(58) *Three Months' Certificate.*—Will you kindly inform me if there is any hospital where, by paying a fee, women over 40 can obtain a certificate after three months' training? I am thoroughly experienced in nursing, but have had no hospital training.—*Pauline.*

(59) *Feeding Bottles.*—Which is the best sort of feeding bottle?—*Sister, Children's Hospital.*

(60) *Training.*—Kindly give me the address of institution for training (lady) nurses for children for permanency in families?—*Barcestree.*

(61) *Local Government Board.*—What provincial hospitals have to obtain the sanction of the Local Government Board to nursing appointments?—*U. A.*

### Answers.

(57) *Training (Monthly Nurse).*—Both midwives and monthly nurses are trained at the East-end Mothers' Home, 396, Commercial Road, E. You can get there easily by tram from Aldgate. For terms write to the Lady Superintendent, Miss B[illegible]nfield.

(58) *Three Months' Certificate (Pauline).*—We do not know of any general hospital which gives a three months' certificate, nor should we attach any value to such a document. It is unfortunate that you have not been able to secure a thorough training before this, but still, in the capacity of companion or attendant on a chronic invalid, you will find congenial work. You must be well suited for this, as you say you are "really an excellent nurse, healthy and cheerful."

(59) *Feeding Bottles (Sister, Children's Hospital).*—There are several very good varieties of the boat-shaped feeding bottle. Some have glass screws, and are easily kept clean. You should pay a visit to one of the good firms and see for yourself. The long tubes should never be used, and even with the others great care is needed to keep them pure. The danger of your plan of leaving the baby to feed itself lies in the probability that this will not be done. The bottle will remain half full, and the child half satisfied. Time devoted to feeding a delicate baby is well spent, not, as you seem to think, wasted. If you cannot get the babies in your ward properly and punctually fed tell the matron so, and she will give you extra help.

(60) *Training (Barcestree).*—Norland Institute, Norland Square.

(61) *Local Government Board (U. A.)*—Workhouse hospitals or infirmaries which are under the Poor Law.

### A Suggestion for Uniform.

By Nurse Bessie.

The accompanying sketch shows a new design for a nurse's uniform which may be an improvement on the present costume of nurses. The dress sketched is intended to be worn with no band to confine it at the waist, and can be fashioned in various ways, either gathered or pleated into the collar, smocked, or gathered into a plain yoke.

The apron band should be worn just low enough to allow of the free movement of the arms, and can be secured by two straps crossing both at the back and front, by which the apron can be kept firmly in place without fitting tightly anywhere. If the straps at the front should be disliked the apron could be made pinafore shape to fit up round the neck, and be secured in place by a sash of some soft white material worn just below the arms and tied in bows with

Nurse Bessie's Ideal.

long ends behind. It would in that way make a comfortable and not ungraceful dress, and with suitable underclothing corsets could be altogether dispensed with. All pressure and weight would thus be entirely removed from the waist. If the wearing of corsets is unnatural and unhealthy for all women it is particularly so for nurses, the strain of constant heavy work requiring perfect freedom not only of muscles, but also of lungs, heart, and digestive organs. Many say they find corsets "a great support." That is due partly to custom and partly to lack of development of the muscles and the consequent weakness needing support. The discarding of corsets could hardly be advised for nurses who have worn them during several years of hard work. Probationers who are beginning their training, or have only worked for a short time, should, if possible, adopt a dress which will not require corsets to ensure comfort and fit. They would in a short time derive great benefit from the change, and would probably escape that trouble so common among nurses of "indigestion." I shall be glad to know what other nurses think of my suggestion?

### The Book World for Women and Nurses.

[We invite Correspondence, Criticism, Enquiries, and Notes on Books likely to interest Women and Nurses. Address, Editor, The Hospital (Nurses' Book World), 428, Strand, W.C.]

Personal Hygiene. By Mrs. Ada S. Ballin. (F. J. Rebman, 11, Adam Street, Strand, London, 1895.)

In giving this "treatise on personal health" to the public Mrs. Ballin has accomplished a useful work. We wish every householder would study the practical suggestions and hints contained in the first chapters on how to choose a house and upon drainage and water suppy, and other domestic matters. The author is probably not far wrong in saying that some women decide the important question of taking the lease of a house in less time than they would require to choose a dress, regardless as to whether "the chimneys smoke, the roof leaks, and the bath in the smart-looking bath-room communicates directly with the main drain, and is a perfect contrivance for admitting typhoid fever and diphtheria into the house." The directions as to clothing and food are clear and sensible, and the chapter on "Rest" is one which might be read with advantage by many a "housemother." Other subjects dealt with are exercise and recreation, baths, digestion, &c., and the final chapter treats of the precautions to be taken in cases of infectious disease. We think Mrs. Ballin expresses here a somewhat excessive faith in "Sanitas" preparations as disinfectants. On page 207 the following direction is given: "For all purposes one cannot do better than use the 'Sanitas' disinfectant preparations, using the scrubbing soap for the floor, and for cleansing clothes and utensils, the toilet soap for the nurse's and patient's ablutions, the powder to scatter about the house and disinfect the patient's excretions, and the fluid for other purposes." We should suggest the substitution of the word "carbolic," that being the most reliable disinfectant for such purposes The book is one which, if studied as it deserves, should lead to a far better general understanding of the first principles of hygiene.

In Sickness and Health. Good Morning and Good Night. Morning and Evening Readings for Children. By the author of "Beneath the Banner." (Price 1s. Cassell and Co., Limited).

This is a book which only requires to be known to become an established favourite in the nursery. It is built up of attractive little stories, presented in just such a way as to take the fancy of a child and provoke a healthy emulation of noble actions; and the words of admonition are so skilfully linked with the characters that all suspicion of moralising is averted. There are just those little home truths pleasantly expressed which many mothers long to put before their children without ever finding the opportunity, and a few minutes daily in company with this little scarlet volume would exactly meet the need and open the way for those intimate talks which help far more than preachments to build up character. A characteristic anecdote of Sir Andrew Clark is worth quoting. "A young man who went to consult the great doctor, Sir Andrew Clark, after telling him about his illness, said: "I find work such a trouble just now, and it is so difficult to do things which before were quite easy." "Ah," said the doctor, "when you find a difficulty in making up your mind to do a thing you must say to yourself, 'I'll do it if I die.'"

Through the Long Night Watch. A Manual for Nurses engaged in Night Nursing. By M. L. B. Privately printed. To be obtained (post free 10d.) from M. L. B., 25, Norfolk Crescent, Hyde Park, W.

We think many nurses engaged in private nursing may find this unpretentious little book helpful and suggestive. It contains short prayers and ejaculations for both nurse and patient, and suggests hymns and passages of Scripture for repeating by the bedside. The short offices to be said before and after laying out the dead are specially good. One great merit of the compilation is the extreme brevity of the devotions, dictated, no doubt, by the personal experience of Nurse B. None but those familiar with illness can understand the inability resulting in rapid exhaustion on the part of the invalid to follow out a train of thought, or listen even for a few minutes to regular devotional reading.

Tuxter's Little Maid. By G. B. Burgin. (Messrs Cassell.) Price 6s.

This is one of the prettiest and pleasantest stories which we have read for a long while. The delicate art displayed in its construction is almost lost sight of in the apparent simplicity of a narrative in which the reader's interest never for one moment flags. The description of the Little Maid's first introduction to Tuxter is so quaint and so graphically told that we are inclined to think it the gem of the book, until we find the very next chapter contains another scene which rivals it. The book is full of humour of so clean and wholesome a kind that we lay it down regretting that the pleasant ending comes so soon. We cordially recommend our readers to judge for themselves of the clever word-painting which makes the incongruous elements of Tuxter's household living realities.

"Yuletide"—Cassell's Christmas Annual—is exception ally well got up this year, both as to its illustrations and literary contents. Some of the pictures are tastefully reproduced in colour. The opening story, "IA," by "Q.," occupies a third of the book; this is followed by "King Arthur; a Tale of Damp Ghosts." Mr. Max Pemberton has a farce in one act, and "Fancy Land" charmingly set in designs by Miss Gertrude Bradley. There are many other items besides these in "Yuletide," which certainly is a remarkably cheap shillingsworth of entertainment.

### The Princess Maud Marriage Present Fund.

Nurses sending contributions to this Fund are requested to write outside the envelope in the left-hand corner the words "Princess Maud," as this will save considerable trouble in dealing with the correspondence. Nurses are reminded that all letters on this subject should be addressed to the Manager of The Hospital, 428, Strand, London, W.C., and not to the office of the Pension Fund.

Amount previously acknowledged ... £49 16s. 0d.

Sixth List.

| | s. | d. | | s. | d. |
|---|---|---|---|---|---|
| S. Crisford ... ... ... | 1 | 0 | F. S. N. ... ... ... | 1 | 0 |
| Policy No. 86 ... ... | 1 | 0 | A. F. Kerr ... ... ... | 1 | 0 |
| F. G. Davis... ... ... | 1 | 0 | A. Griffiths... ... ... | 1 | 0 |
| A. C. Holmes ... ... | 1 | 0 | Policy No. 3,145 ... | 1 | 0 |
| S. A. Box ... ... ... | 2 | 0 | M. Wright ... ... ... | 1 | 0 |
| M. J. Tirpe ... ... | 1 | 0 | E. D. Kelsey ... ... | 1 | 0 |
| Nurse Ashford ... ... | 1 | 0 | A. J. Brunsden ... ... | 1 | 0 |
| K. Saczkovic ... ... | 1 | 0 | E. Hoskier ... ... ... | 1 | 0 |
| Policy No. 2,110 ... | 1 | 0 | R. S. ... ... ... ... | 1 | 0 |
| Policy No. 2,564 ... | 1 | 0 | M. Blayney ... ... | 1 | 0 |
| Policy No. 4,027 ... | 1 | 0 | Policy No. 658 ... ... | 1 | 0 |
| F. Cupitt ... ... ... | 1 | 0 | E. Smith ... ... ... | 1 | 0 |
| C. Boulter ... ... ... | 1 | 0 | Nurse Amor ... ... | 1 | 0 |
| A. W. Hutchinson ... | 1 | 0 | E. Amor ... ... ... | 1 | 0 |

Received per Royal National Pension Fund.

| | s. | d. | | s. | d. |
|---|---|---|---|---|---|
| A. Wood ... ... ... | 1 | 0 | Nurse Burgess ... ... | 1 | 0 |
| J. M. Thwaites ... ... | 1 | 0 | E. M. Walton ... ... | 1 | 0 |

### Wants and Workers.

[The attention of correspondents is directed to the fact that "Helps in Sickness and to Health" (Scientific Press, 428, Strand) will enable them promptly to find the most suitable accommodation for difficult or special cases.]

A lady is willing to give an invalid carriage (in good condition) for the use of a child of seven. Apply by letter only, with particulars of case, to H. F. Gethen, The Hospital Office, 428, Strand.

## Figures and Tables

**Figure f1:**